# A New Strain Collection for Improved Expression of Outer Membrane Proteins

**DOI:** 10.3389/fcimb.2017.00464

**Published:** 2017-11-07

**Authors:** Ina Meuskens, Marcin Michalik, Nandini Chauhan, Dirk Linke, Jack C. Leo

**Affiliations:** ^1^Section for Evolution and Genetics, Department of Biosciences, University of Oslo, Oslo, Norway; ^2^Interfaculty Institute for Biochemistry, Eberhard Karls University, Tübingen, Germany

**Keywords:** outer membrane, β-barrel protein, recombinant protein expression, P1 transduction, production strain

## Abstract

Almost all integral membrane proteins found in the outer membranes of Gram-negative bacteria belong to the transmembrane β-barrel family. These proteins are not only important for nutrient uptake and homeostasis, but are also involved in such processes as adhesion, protein secretion, biofilm formation, and virulence. As surface exposed molecules, outer membrane β-barrel proteins are also potential drug and vaccine targets. High production levels of heterologously expressed proteins are desirable for biochemical and especially structural studies, but over-expression and subsequent purification of membrane proteins, including outer membrane proteins, can be challenging. Here, we present a set of deletion mutants derived from *E. coli* BL21 Gold (DE3) designed for the over-expression of recombinant outer membrane proteins. These strains harbor deletions of four genes encoding abundant β-barrel proteins in the outer membrane (OmpA, OmpC, OmpF, and LamB), both single and in all combinations of double, triple, and quadruple knock-outs. The sequences encoding these outer membrane proteins were deleted completely, leaving only a minimal scar sequence, thus preventing the possibility of genetic reversion. Expression tests in the quadruple mutant strain with four test proteins, including a small outer membrane β-barrel protein and variants thereof as well as two virulence-related autotransporters, showed significantly improved expression and better quality of the produced proteins over the parent strain. Differences in growth behavior and aggregation in the presence of high salt were observed, but these phenomena did not negatively influence the expression in the quadruple mutant strain when handled as we recommend. The strains produced in this study can be used for outer membrane protein production and purification, but are also uniquely useful for labeling experiments for biophysical measurements in the native membrane environment.

## Introduction

The envelope of Gram-negative bacteria such as, *Escherichia coli* consists of two membranes, the inner and the outer membrane. This double membrane system protects the bacteria from environmental insult and makes them resistant to many antibiotics and host immune defenses, but allows the efficient uptake of nutrients. The outer membrane is permeable to small hydrophilic molecules due to the presence of porins. Porins, and almost all other transmembrane outer membrane proteins (OMPs), are composed of a transmembrane β-barrel domain (Fairman et al., 2011). β-barrels consist of an antiparallel β-sheet that closes in on itself; the proteins thus adopt a cylindrical shape, with hydrophobic residues facing the membrane environment and mostly hydrophilic residues lining the inside of the β-barrel, which in the case of porins acts as an aqueous channel permitting the diffusion of water and other nutrients through the outer membrane (Delcour, 2009). Other OMPs act as secretion pores, transporting a variety of macromolecules across the outer membrane, such as, lipopolysaccharide (Dong et al., 2014), biofilm matrix components (Hufnagel et al., 2015), other proteins (Chagnot et al., 2013), or, in the case of autotransporters, parts of the same polypeptide chain (Leo et al., 2012). OMPs are further involved in such functions as self-recognition (Aoki et al., 2005, 2008), protein hydrolysis (Haiko et al., 2009), and virulence (Monteiro et al., 2016).

All β-barrel OMPs in Gram-negative bacteria are homologous (Remmert et al., 2010), and follow a conserved route of membrane insertion. OMPs are transported across the inner membrane via the Sec machinery in an unfolded conformation (Walther et al., 2009b). In the periplasm, chaperones such as, SurA, Skp, and DegP help to keep the OMPs in an unfolded state (Goemans et al., 2014). Insertion of OMPs into the outer membrane is accomplished by the β-barrel assembly machinery or BAM complex (Bakelar et al., 2016; Gu et al., 2016; Han et al., 2016). A recent study has shown that OMPs are inserted into the outer membrane at discreet sites near the cell center and move laterally toward the cell poles (Rassam et al., 2015). As the periplasm is devoid of adenosine triphosphate and ionic gradients cannot be maintained across the outer membrane, the energy for insertion into the outer membrane must be provided by the folding of the β-barrel itself (Moon et al., 2013).

Insertion of OMPs is thus dependent on the two constitutive membrane insertase/translocase systems, the Sec, and the BAM. For efficient recombinant production of properly folded OMPs, sufficient capacity is required for both systems to process the additional burden of heterologously expressed protein. When the BAM copy number is reduced, OMPs are inefficiently integrated into the outer membrane, though cell viability is not significantly affected (Aoki et al., 2008). Thus, under OMP over-expression conditions, the BAM may become congested, resulting in a bottleneck for efficient OMP integration. In addition to misfolding, this may also lead to induction of the envelope stress response, and thus indirectly to induction of protease expression (Alba and Gross, 2004) including the periplasmic protease DegP (Grosskinsky et al., 2007).

The Sec system is also prone to saturation, based e.g., on observations that, in some over-expression conditions, cytosolic inclusion bodies are formed where the signal peptide was not properly processed, or periplasmic inclusion bodies are observed due to follow-up problems of improper processing (Georgiou and Segatori, 2005). Over-expression of inner membrane proteins leads to accumulation of cytoplasmic inclusion bodies and aggregates, but also reduces the amount of proteins secreted into the periplasm and outer membrane (Wagner et al., 2007). Congestion of the Sec machinery further affects cell viability and the maximum rate at which heterologous OMPs can be transported into the periplasm (Schlegel et al., 2013). The Sec machinery also transports soluble periplasmic proteins, lipoproteins, integral inner membrane proteins, and several types of secreted proteins in addition to OMPs (Kudva et al., 2013). Therefore, for over-expression of heterologous OMPs in *E. coli*, it would be advisable to knock out abundant but non-essential OMPs to relieve some of the burden on the BAM and Sec machineries. In addition, removing these abundant proteins from the membrane leaves more space for recombinant proteins, potentially influencing the maximum yield per cell; limited membrane area can be a bottleneck for the over-production of membrane proteins (Arechaga et al., 2000; Wagner et al., 2006).

Koebnik and coworkers have previously developed such a set of strains derived from the common expression strain BL21(DE3) (Prilipov et al., 1998b). These strains lack one or more of the most abundant OMPs: OmpA, OmpC, OmpF, or LamB (maltoporin). We have successfully used some of these strains for expression and purification of OMPs, especially BL21 Omp2 and BL21 Omp8 (Wollmann et al., 2006; Arnold et al., 2007; Leo et al., 2011; Mikula et al., 2012; Oberhettinger et al., 2012; Shahid et al., 2012), but also for NMR experiments using native membranes (Shahid et al., 2015). However, in our hands these strains have proven to be genetically unstable and prone to sudden lysis, possibly due to mobilization of the Tn*5* transposon under stress conditions, used in generating these knock-out strains (Prilipov et al., 1998b; Supplementary Figure 1).

In this work, we present a series of knock-out strains lacking one, two, three, or all four of the genes encoding the proteins OmpA, OmpC, OmpF, and LamB. These strains are designed for use in over-expression of recombinant OMPs, and are equivalent to some of the strains produced earlier (Prilipov et al., 1998b). However, our series is more complete than that produced by Prilipov et al. and we used a different strategy to produce our knock-outs resulting in genetically more stable strains. Particularly, we have not observed spontaneous lysis of our quadruple knock-out strain lacking all four abundant OMPs (similar to the Omp8 strain produced earlier) when handled as we recommend. We also demonstrate that the quadruple mutant strain shows improved levels of four test proteins in the outer membrane compared to the BL21(DE3) parent strain.

## Materials and methods

### Bacteria, media, and growth conditions

The *E. coli* strains produced in this work are derivatives of the commonly used expression strain BL21(DE3) (Studier and Moffatt, 1986). In addition to being widely utilized for expression, this strain also lacks the outer membrane protease OmpT, which we reasoned would be beneficial for over-expression of OMPs in particular. For transductions, we used the generally transducing bacteriophage P1 *vir*. The donor strains harboring the kanamycin cassettes for gene deletion were from the Keio collection (Baba et al., 2006). The K-12 reference strain was BW25113 (Datsenko and Wanner, 2000).

Bacteria were grown in lysogeny broth (LB) unless stated otherwise. For most experiments, we used the “Lennox” formulation: 10 g tryptone, 5 g yeast extract, and 5 g NaCl per liter. In the text below, we mean this formulation when referring to LB. For growth curves, we also used the “Miller” formulation (10 g tryptone, 5 g yeast extract, and 10 g NaCl per liter)—we refer to this as LB-Miller. Where necessary, media were supplemented with kanamycin (kan) or ampicillin (amp) at 25 μg/ml and 100 μg/ml, respectively. SOC medium was used after transformations as described elsewhere (Hanahan, 1983). As a defined medium, we used the minimal medium M9 (Miller, 1972) supplemented with glucose 0.2% (w/v) and 18 amino acids (all except cysteine and tyrosine) at 0.1 mg/ml each. Bacteria were usually grown at 37°C, unless harboring the plasmid pCP20, in which case they were grown either at 30°C for plasmid maintenance or 42°C to cure the plasmid. Some of the *omp* deletion strains, such as, the quadruple mutant BL21ΔABCF, grew significantly better at 30°C than at 37°C, and these were thus propagated at 30°C.

### Plasmids

Plasmid pCP20 encoding the FLP recombinase (Flippase) was used for excising the kanamycin cassette after transduction into the knock-out strains (Cherepanov and Wackernagel, 1995). For λ red recombination, we employed the plasmid pKD46 (Datsenko and Wanner, 2000). The expression constructs used for testing our strains have been described previously: pET3b containing the genes encoding *ompX* and variants thereof (Arnold et al., 2007), pASK-IBA2 with *Yersinia enterocolitica* YadA membrane anchor domain (YadAM) (Wollmann et al., 2006) or Intimin (Oberhettinger et al., 2012). pET3b-OmpX was also modified for the purpose of this study by inserting a double haemagglutinin (HA)-tag with GSG linkers (GSGYPYDVPDYAGSGYPYDVPDYAGSG) in the position between S53 and S54 of OmpX for easier detection (pET3b-OmpX-HA). The insertion was created by site-directed mutagenesis (Byrappa et al., 1995) using the primers given in Table 1.

**Table 1:** Primers used in this study.

**COLONY PCR**		
**Gene**	**Direction**	**Sequence (5′ → 3′)**
*ompA*	Forward	ATTTTGGATGATAACGAGGCGCAAAAAATG
*ompA*	Reverse	GAACTTAAGCCTGCGGCTGAGTTAC
*lamB*	Forward	AAAAGAAAAGCAATGACTCAGGAGATAGAATG
*lamB*	Reverse	GGTTTTGCTATTACCACCAGATTTCCATCTG
*ompF*	Forward	AGGTGTCATAAAAAAAACCATGAGGGTAATAAATAAT
*ompF*	Reverse	GAGGTGTGCTATTAGAACTGGTAAACGATACC
*ompC* (common)	Forward	CAATCGGTGCAAATGCCAGATAAGACAC
*ompC* (*E. coli* K-12)	Forward	GCAAATAAAGGCATATAACAGAGGGTTAATAACATG
*ompC*	Reverse	ATATCAATCGAGATTAGAACTGGTAAACCAGACC
**MUTAGENESIS**		
**Product**	**Direction**	**Sequence (5′ → 3′)**
HA-OmpX	Forward	GTTATCCATACGACGTACCTGATTACGCAGGTTCTGGGTCTGGTGACTACAACAAAAACCAG
HA-OmpX	Reverse	CTGAGCCCGCATAATCCGGAACATCATACGGGTAACCAGAACCGCTTGCAGTACGGCTTTTCTC

### P1 phage transduction

P1 phage transduction was performed essentially as described (Thomason et al., 2007). Briefly, the *E. coli* strains from the Keio collection were grown in LB medium to an optical density at 600 nm (OD_600_) of 1.0 and infected with P1 *vir* phages at various dilutions (e.g., 10^−5^, 10^−6^, 10^−7^), mixed with 3 ml liquid top agar and poured onto pre-warmed LB plates. The following day, a semi-confluent plate was chosen and the top agar scraped off. This was mixed with 2 ml LB medium supplemented with a drop of chloroform, vortexed for 2 min and centrifuged for 10 min at 5,000 × g. Another drop of chloroform added to the supernatant, which was then stored at 4°C. The parent strain *E. coli* BL21 Gold (DE3) was infected with dilution series of each P1 phage lysate and the titer of the lysates was calculated from the number of plaques formed on the plates.

For the transduction experiments, *E. coli* BL21 Gold (DE3) and derivatives were grown in LB medium overnight, diluted 1:100 in the morning and grown until an OD_600_ of ~1.0, and then supplemented with 10 mM CaCl_2_ and mixed the P1 phage lysate harboring the kan cassette specific for the desired knock-out at a multiplicity of infection (MOI) of 0.5, assuming 10^9^ bacteria/ml culture. After incubating for 20 min at 37°C, the infection was stopped by addition of 100 mM sodium citrate pH 5.5. The mix was centrifuged for 2 min at 5,000 × *g*. The pellet was washed in LB medium supplemented with 100 mM sodium citrate and centrifuged as before. This washing step was repeated twice and then the bacteria were incubated for ~1 h at 37°C in LB containing 100 mM sodium citrate. Finally, the bacteria were centrifuged for 2 min at 4,000 × *g* diluted in 100 μl LB medium and streaked out on LB agar plates supplemented with kanamycin and 10 mM sodium citrate.

### Introduction of the kan cassette by λ red recombination

To delete the *ompF* locus, we amplified the FLP recognition target (FRT)-kanamycin cassette flanked by sequence directly outside the sequence coding for OmpF, using the Keio collection Δ*ompF* strain and the same primer sequences described in that study (Baba et al., 2006). Insertion of the kan cassette was achieved by λ red recombination, essentially as described (Datsenko and Wanner, 2000): the plasmid pKD46 was transformed into recipient strains by electroporation, and transformants were selected for by plating on ampicillin and growing at 30°C. To introduce the kan cassette into the *ompF* locus, the pKD46-containing bacteria were grown to mid-log phase at 30°C, at which time the λ red genes were induced by the addition of 1 mM L-arabinose. After 1 h of induction, the cells were harvested and made electrocompetent. One hundred nanograms of PCR product was transformed into the cells by electroporation, after which cells were allowed to recover for 1 h at 30°C in SOC medium supplemented with 1 mM L-arabinose. Transformants were then selected for by growing on LB with kanamycin at 30°C. To remove pKD46, bacteria were grown on LB + kan at 37°C, and then tested for ampicillin sensitivity. Insertion of the kan cassette into the *ompF* locus was verified by colony PCR.

### Excision of the kan cassette

For excision of the kan cassette, kan-resistant transductants were transformed with the conditionally replicating plasmid pCP20 encoding the FLP recombinase by electroporation, following the procedure suggested by Baba et al. (2006). After electroporation the cells were quickly mixed with 1 ml SOC medium and incubated for 1 h at 30°C. The bacteria were then plated on LB agar plates with ampicillin and incubated overnight at 30°C. To cure pCP20, one amp-resistant colony was streaked out onto an LB agar plate and incubated at 42°C overnight. To screen for mutants strains, colonies were streaked out on LB plates containing kan, amp, and no antibiotic, respectively, using a grid, and then incubated at 37°C overnight. Clones sensitive to both kan and amp were chosen, and correct deletions were verified using colony PCR.

### Colony PCR

For verification of the right gene deletions in our mutants PCR with primers specific for the upstream and downstream region of the gene to be deleted was used. The primers sequences are given in Table 1. Colony PCR was performed using Taq polymerase (New England Biolabs) and 20 pmol of primer per reaction. A typical colony PCR program was as follows: initial denaturation for 3 min at 94°C, followed by 25 cycles of denaturation (30 s at 94°C), annealing (20 s 50°C), and extension [1.5 min (or 5 min with *ompC* common primers) at 70°C]. After a final extension of 5 min at 70°C, samples were mixed with loading buffer and applied to a 1% agarose gel (0.8% for *ompC* common primers). The primer pair used amplified the coding sequences of each locus are indicated in Table 1.

### Outer membrane preparations

Outer membrane isolations were performed essentially as described in Leo et al. (2015). Briefly, 20 ml of an overnight culture at OD_600_ 1.0 were pelleted and washed with 10 mM HEPES buffer at pH 7.4. To promote lysis, 0.1 mg/ml lysozyme was added, along with MgCl_2_ and MnCl_2_ to 10 mM and a pinch of DNase I (Sigma). The cells were then disrupted using a bead beater (SpeedMill Plus from Analytik Jena, Germany). The lysates were centrifuged at 15,600 × *g* for 30 s in a tabletop centrifuge and the supernatant was then moved to a fresh 2 ml microcentrifuge tube and centrifuged at 15,600 × *g* for another 30 min. The supernatant was decanted and the brownish membrane pellet resuspended in 400 μl 10 mM HEPES pH 7.4 with 1% (w/v) *N*-lauroyl sarcosine. The inner membranes were solubilized at room temperature (RT) for 30 min. Following this, the tubes were centrifuged at 15,600 × *g* for 30 min to pellet the outer membrane. The pellet was washed with 10 mM HEPES pH 7.4 and then resuspended in 30 μl HEPES buffer. For SDS-PAGE, 10 μl of 4 × non-reducing SDS sample buffer was added. Fifteen percentage SDS-PAGE gels were used for experimental verification of the knockout mutants at the protein level. For Coomassie G-250 (colloidal) staining of polyacrylamide gels, 12 μl of the outer membrane samples were used. For silver staining (Nesterenko et al., 1994), 8 μl of the outer membrane prep samples were run in a polyacrylamide gel and for Western blots 6 μl of the outer membrane prep samples were used.

### Growth curves

To draw growth curves, starter cultures were grown in 5 ml LB or supplemented M9 medium and the OD_600_ values were measured. The bacteria were then diluted to an OD_600_ value of 0.01 and 5 μl of this suspension was added to 200 μl of medium (LB, LB-Miller, or supplemented M9) in a sterile microtiter plate. For blanks, no bacteria were added. The plates were sealed with a BreathEasy® membrane (from Sigma-Aldrich). The plates were incubated in a Biotek Synergy plate reader with controlled temperature and orbital rotation at the “slow” setting; absorbance at 600 nm was recorded at 20-min intervals. For plotting, values from four biological replicates were averaged.

### Aggregation assays

For sedimentation assays, an overnight culture was diluted 1:100 in fresh LB (total volume 10 ml). The cultures were grown with shaking at 30°C in a flask till late log phase (OD_600_ ~1.0), at which point MgCl_2_ or CaCl_2_ were added to 10 mM to some of the cultures. After a further hour of incubation at 30°C with shaking, the bacteria were transferred carefully to 18 mm tubes so as not to disrupt any floccules. The tubes were then incubated statically at RT. One hundred and fifty microliter samples were taken from the very top of the cultures at 5-min intervals for 20 min and the absorbance at 600 nm was measured using a microcuvette (light path 1 cm). To estimate autoaggregation, the absorbance at each time point was compared to the absorbance at time point zero and expressed as a percentage:

$$({\text{A}}_{\text{t}}{\;}^{*}\;100)/{\text{A}}_{0},$$

where A_t_ is the absorbance at the relevant time point and A_0_ is the absorbance at time point zero. For plotting, three biological replicates were used.

For photography, 5 ml cultures were grown to late log phase (OD_600_ ≈ 0.8) and MgCl_2_ or CaCl_2_ were added as above. After a further hour of shaking, the tubes were incubated statically at RT and photographed after 20 min and 2 h.

### Recombinant protein expression and detection

For inducing recombinant protein production, plasmids encoding the test proteins were transform into the quadruple mutant strain (BL21ΔABCF). For production, a 5 ml overnight culture of a transformed clone was diluted 1:100 in fresh LB and grown at 30°C till mid-log phase (OD_600_ ~0.5). The culture was then induced with either isopropyl thiogalactoside (at 1 mM) or anhydrotetracycline (at 50 ng/ml). The cultures were incubated at 30°C for a further 2 h, after which the cells were harvested and outer membranes were isolated. The OMPs were separated by SDS-PAGE. Over-expressed YadAM, OmpX, and its duplicated variant OmpX88 were detected by colloidal Coomassie G-250 staining. Overexpression of a HA-tagged variant of OmpX and Intimin was detected by immunoblotting. The proteins were transferred to a 0.45 μm polyvinylidine difluoride membrane (Thermo Scientific) using a semi-dry transfer unit (Hoefer TE70X). The membranes were blocked with 2% skimmed milk powder dissolved in phosphate-buffered saline (PBS; 20 mM sodium phosphate pH 7.4, 150 mMNaCl) for 1 h at RT or overnight at 4°C. This was followed by incubation for an hour with primary antibodies; 1:2,000 dilution of rabbit anti-HA tag antibody (for OmpX-HA) and 1:5000 dilution of rabbit anti-Intimin antibody (Oberhettinger et al., 2012). The membrane was washed three times with PBS+0.05% Tween20 followed by incubation with a 1:10,000 dilution of goat anti-rabbit IgG horseradish peroxidase-conjugate (Santa Cruz Biotech) in PBS+2% skimmed milk powder for an hour. The membrane was washed three times with PBS+0.05% Tween20. The bands were detected using enhanced chemiluminescence (Pierce ECL western blotting substrate) and a Kodak 4000R Image station.

### Whole-cell ELISAs

For quantitative examination of Intimin and OmpX-HA expression, we performed whole-cell enzyme-linked immunosorbent assays (ELISAs). Bacteria transformed with the corresponding plasmids were grown overnight at 30°C in 5 ml LB medium with ampicillin. The following day, the bacteria were diluted 1:10 in fresh medium (5 ml, with amp) and grown till mid-log (OD_600_ ~0.5; about 2 h), at which time they were induced with anhydrotetracycline or isopropyl thiogalactoside as in the section on Recombinant Protein Expression and Detection. The bacteria were grown at 30°C for another 2 h. The OD_600_ of the cultures was measured and the bacteria were diluted in PBS to an OD_600_ value of 0.2. One hundred microliters of this suspension were applied to the wells of a polystyrene microtiter plate and bacteria were allowed to adhere to the surface of the well for 1 h at RT. The wells were washed three times with 200 μl washing buffer [PBS + 0.1% bovine serum albumin (BSA, from VWR)] and then blocked for 1 h with PBS + 2% BSA. The wells were washed once with 200 μl washing buffer, and 100 μl of the primary antibody diluted into blocking buffer was applied. The antibodies were the same as in the section on Recombinant Protein Expression and Detection, anti-HA (1:2,000), and anti-Intimin (1:1,000). After an hour's incubation, the wells were washed three times as above, and the secondary antibody (anti-rabbit-HRP, from Agrisera) was added, diluted 1:2,000 in blocking buffer. The plate was incubated for 1 h at RT, after which the wells were washed three times as above. Detection was performed using the colorimetric HRP substrate ABTS (ThermoScientific) according to the manufacturer's instructions. After color development (40 min) the reactions were stopped with 1% SDS and absorbances were recorded at 405 nm.

## Results

### Production of knock-out strains

To avoid the problems we had encountered with the strains from Prilipov et al. we pursued a different strategy in making our knock-out strains. We decided to delete the entire coding sequences for the four genes encoding the OMPs lacking in the Omp8 strain of Prilipov et al. namely *ompA, ompC, ompF*, and *lamB* (Prilipov et al., 1998b). We reasoned that this strategy would prevent the possibility of a genetic reversion, thus improving the genetic stability of the newly generated knock-out strains. BL21 strains probably do not express *ompC* naturally, due to an insertion element-mediated deletion of the upstream region of the *ompC* locus, including the signal peptide of OmpC (Pugsley and Rosenbusch, 1983; Studier et al., 2009; Han et al., 2012). Nevertheless, we decided to delete the entire *ompC* coding sequence to fully prevent the possibility of reversion, e.g., by a recombination event restoring a functional signal peptide. To make the deletions, we employed the Keio collection, a set of 3,985 single-gene deletions in *E. coli*, where virtually the entire coding sequence of the deleted genes is replaced by a kanamycin resistance cassette (Baba et al., 2006). This cassette is flanked by sequences targeted by the FLP recombinase; thus, when FLP is supplied in trans, the kan cassette can be excised from the genome leaving only a ~100 bp-long scar sequence.

We produced the single knock-outs in *E. coli* BL21 Gold (DE3) by phage P1 transduction: a phage lysate was produced from the Keio strains harboring the desired OMP gene deletions. This lysate was used to infect the recipient strain, and kan-resistant transductants were then selected for by plating on kanamycin plates. To remove the kan cassette, we introduced the FLP-containing plasmid pCP20 into the kan-resistant bacteria and selected for amp-resistant colonies without the addition of kanamycin. To cure pCP20, clones were plated onto LB (no selection) and grown at 42°C. Clones that were sensitive to both amp and kan were chosen for PCR screening to verify the loss of the kan cassette and the loss of the wild-type allele. Using this strategy, we produced the four individual knock-outs, and then by re-iterating the process, we obtained five double knock-outs, three triple knock-outs, and the quadruple knock-out strain (Table 2).

**Table 2:** Knock-out strains produced in this study.

**Knock-outs**	**Strain**	**Genes deleted**	**Addgene ID**
**SINGLE KNOCK-OUTS**			
	BL21ΔA	*ompA*	102256
	BL21ΔB	*lamB*	102257
	BL21ΔC	*ompC*	102258
	BL21ΔF	*ompF*	102259
**DOUBLE KNOCK-OUTS**			
	BL21ΔAB	*ompA, lamB*	102260
	BL21ΔAC	*ompA, ompC*	102261
	BL21ΔAF	*ompA, ompF*	102262
	BL21ΔBC	*lamB, ompC*	102263
	BL21ΔBF	*lamB, ompF*	102264
	BL21ΔCF	*ompC, ompF*	102265
**TRIPLE KNOCK-OUTS**			
	BL21ΔABC	*ompA, lamB, ompC*	102266
	BL21ΔABF	*ompA, lamB*,*ompF*	102267
	BL21ΔACF	*ompA, ompC, ompF*	102268
	BL21ΔBCF	*lamB, ompC, ompF*	102269
**QUADRUPLE KNOCK-OUT**			
	BL21ΔABCF	*ompA, lamB, ompC, ompF*	102270

*All strains are derived from BL21 Gold (DE3). With the genotype: *E. coli* B F^−^*ompT hsdS*(rB^−^ mB^−^) *dcm*^+^ Tet^r^*gal endA* Hte.*.

We experienced particular problems producing the *ompA*-*ompF* double knock-out and the *ompA*-*ompF*-*ompC* triple knock-out. The *ompA* and *ompF* loci are situated relatively close to each other in the *E. coli* genome (distance ~32 kb; P1 can transduce fragments ~ three times this size); therefore, we often obtained revertants for one mutant while trying to produce the other. To circumvent this problem, we employed a different strategy: we amplified the FRT-kan cassette from the Keio Δ*ompF* strain by PCR, with 50 bp overhangs identical to the sequence flanking the *ompF* coding region, as was used to make the *ompF* mutant in the original Keio collection (Baba et al., 2006). We then produced the BL21ΔAF and BL21ΔACF strains by λ red recombination in the BL21ΔA and BL21ΔAC strains (Datsenko and Wanner, 2000). We then selected for kan-resistant colonies and subsequently removed the kan cassette as described for the transduction experiments. Indeed, this strategy proved successful.

### Verification of the knock-out mutant strains

**Figure 1:**
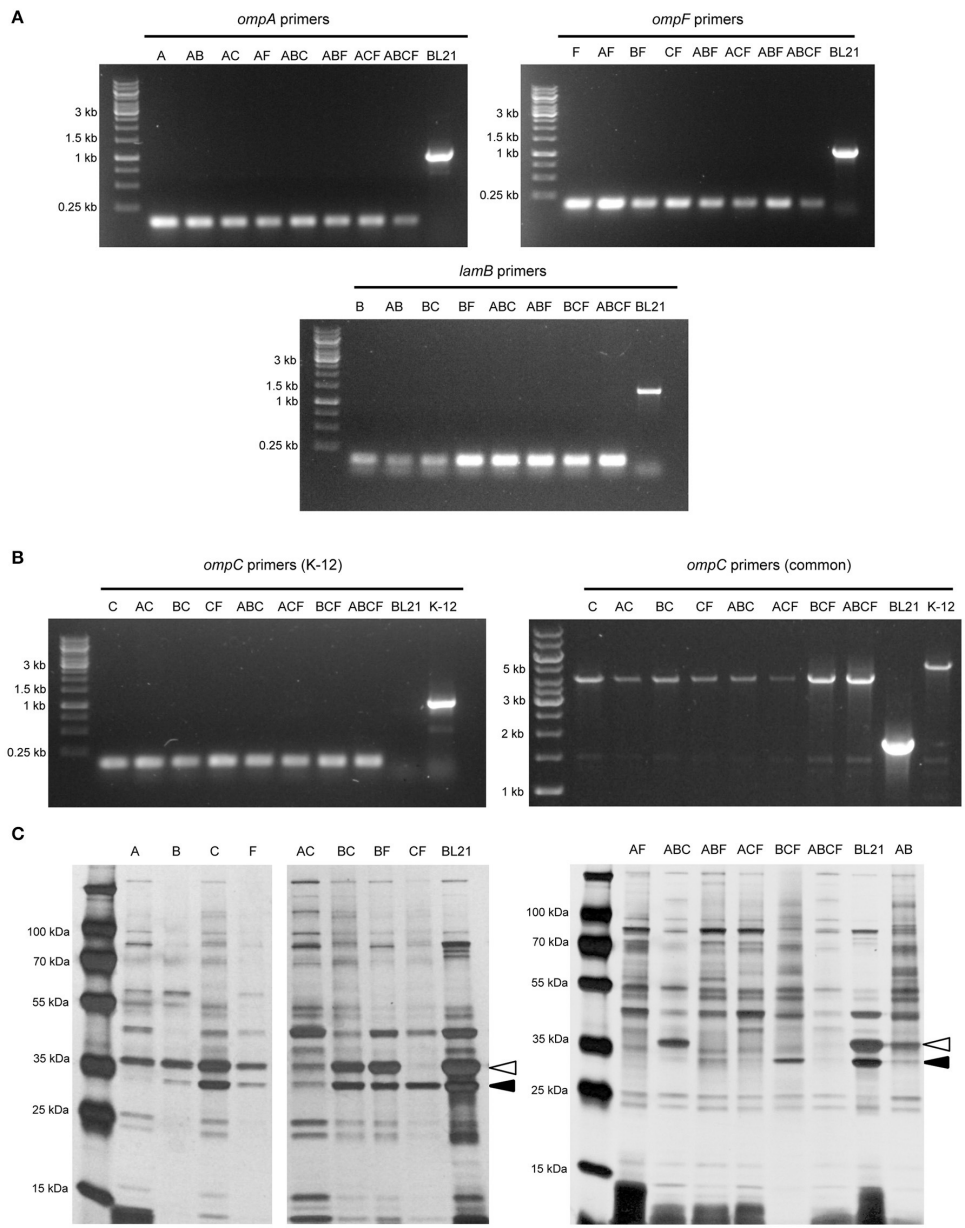
Verification of the BL21 OMP knock-out strains. **(A)** Colony PCR results with primers for *ompA, ompF*, and *lamB* showing that in the mutant strains only a scar sequence (130–150 bp) remains at the locus. BL21 = (DE3) control (expected sizes: *ompA* 1072, *ompF* 1135, *lamB* 1380 bp). **(B)** Colony PCR results of *ompC*. On the left, results using primers specific for the K-12 *ompC* locus are shown, where BL21(DE3) does not give a product and the deletion strains show just a short scar sequence (~150 bp). The expected size for K-12 *ompC is* 1101 bp. On the right, results using common primers amplifying a larger region around the *ompC* locus in both BL21 and K-12. Here, the expected product for BL21(DE3) is 1.9 kb, the size expected for *E. coli* K-12 product is 5.3 kb and for the deletion strains 4.2 kb. **(C)** Silver-stained 15% polyacrylamide gel of BL21 OMP knock-out strains. The positions of OmpA (black arrowhead) and OmpC/F (open arrowhead) bands are indicated for the control (BL21). LamB is poorly expressed in *E. coli* B strains when grown at temperatures above 30°C and in the presence of other carbon sources (Ronen and Raanan-Ashkenazi, 1971), so this protein is not evident in most of the samples. Note that the Δ symbol has been omitted in the figure texts due to space constraints.

To verify that the correct gene had been deleted, we amplified the target coding sequence by colony PCR. Using primers specific for each of the four loci, we could show that the mutant strains had lost the wild-type allele (Figure 1A). The sequences remaining in the deletion mutants correspond to ~150 bp, the size of the scar sequence and flanking regions, indicating that the gene was actually replaced by the kan cassette, which in turn was excised by FLP. The size of the wild-type coding sequence is 1041 bp for *ompA*, 1089 bp for *ompF*, 1104 bp for *ompC*, and 1341 bp for *lamB*. These sizes are consistent with the PCR products obtained from the parent strain BL21 Gold (DE3). For verification of the *ompC* deletion, another forward primer binding downstream the *rcsC* locus was used due to the fact that in *E. coli* BL21 the upstream region of *ompC* is deleted (Studier et al., 2009). Thus, the forward primer amplifying the *ompC* locus in *E. coli* K-12 strains does not produce a product in B strains. The common forward primer, which binds downstream of the *rscC* gene, produces a product in both strains when combined with the same reverse primer used for amplifying the K-12 locus (Table 1). In BL21 Gold (DE3), this results in a product of ~1.9 kb. In K-12, the product also contains the region deleted in BL21 Gold (DE3), so the total length of the product is ~5.3 kb. For the mutants, where the *ompC* coding sequence is deleted within the K-12 context, the product is ~4.2 kb (Figure 1B). These results confirm that the kan cassette had correctly replaced the *ompC* sequence of BL21 Gold (DE3).

In order to show that the PCR-positive knock-out strains lack the corresponding proteins, we prepared outer membrane samples from all the strains and analyzed these by SDS-PAGE and silver staining (Figure 1C). *E. coli* BL21(DE3) was included as a positive sample showing outer membrane protein bands of interest. Maltoporin (LamB) has a size of ~49 kDa. OmpC and OmpF are approximately the same size and run as a single band at ~39 kDa, and OmpA has the smallest size of ~35 kDa. All of these protein bands are visible in the parent strain. The single knock-out strain BL21ΔA shows all bands of interest except the 35 kDa OmpA band indicating that this protein is lacking in this strain. The ΔB strain shows the bands of interest at 35 kDa for OmpA and at 37 kDa for OmpC and OmpF but no band at 49 kDa. As a note, maltoporin is not well expressed in *E. coli* B strains, including BL21, which makes the confirmation of the *lamB* knock-out difficult on the protein level (Ronen and Raanan-Ashkenazi, 1971; Han et al., 2012); thus, the *lamB* knock-outs could only be fully confirmed by PCR. Single *ompC* and *ompF* knock-outs show a reduction in band intensity at 39 kDa, and only the double knock-outs lack the band at 39 kDa completely, suggesting that our strategy of knocking out *ompC* was a reasonable approach to make sure the gene is entirely inactivated. A possible explanation for a residual band at 39 kDa (e.g. for BL21ΔF) is expression of the cryptic porin OmpN (Prilipov et al., 1998a). Taken together, the PCR results and OMP profiles of the strains show that we have successfully deleted the genes encoding the major OMPs, either singly or in all combinations.

### Growth properties of the quadruple knock-out strain BL21ΔABCF

**Figure 2:**
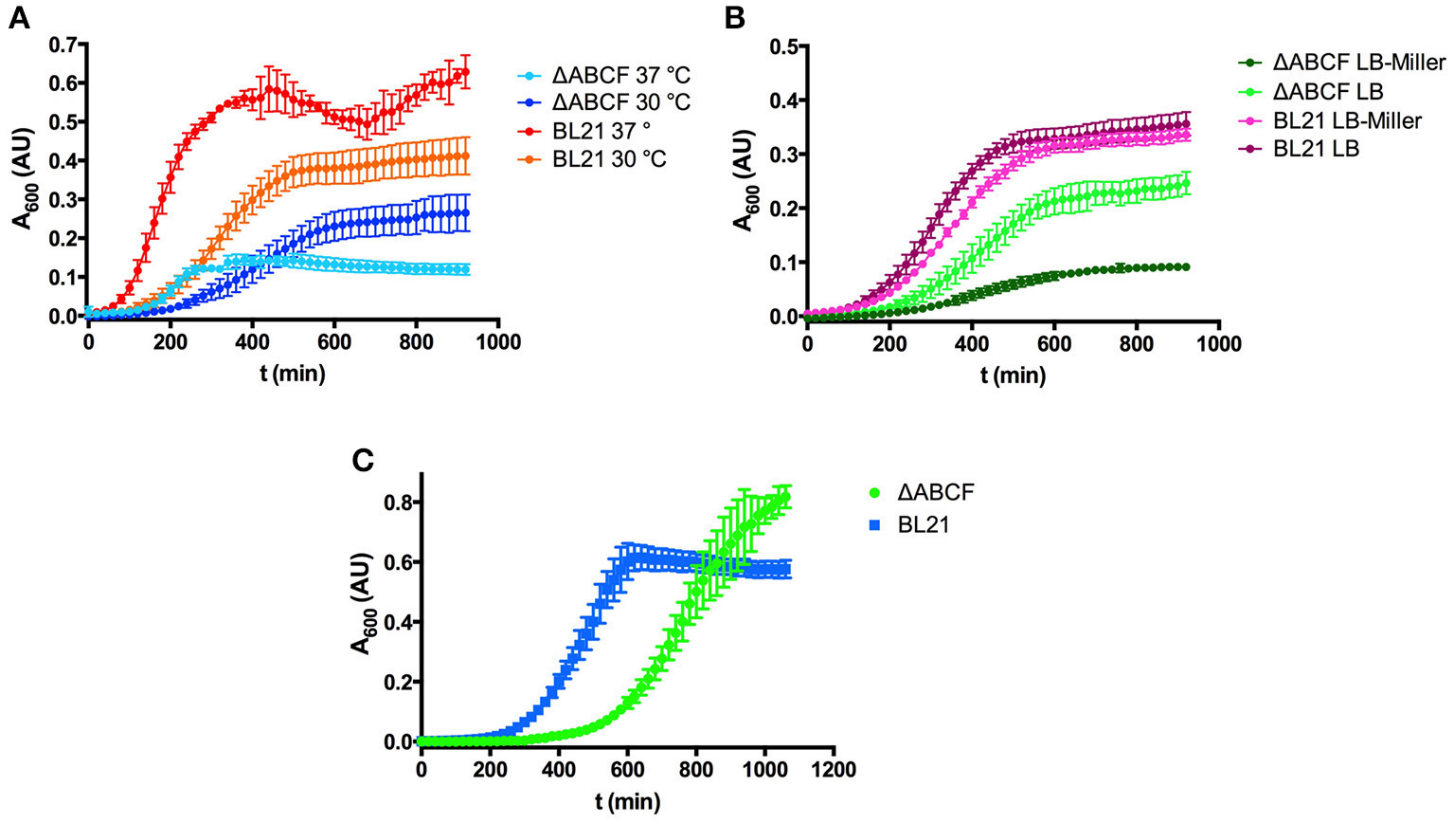
Growth properties of the quadruple mutant BL21ΔABCF. **(A)** Growth of BL21ΔABCF in LB medium at 30 and 37°C. BL21(DE3) is shown for comparison. Data points are the mean of four biological replicates; error bars denote the standard deviation. **(B)** Growth of BL21ΔABCF in LB (0.5% NaCl) and LB-Miller (1% NaCl) medium at 30°C. BL21(DE3) is shown for comparison. Data points are the mean of four biological replicates; error bars denote the standard deviation. **(C)** Growth of BL21ΔABCF in supplemented M9 medium at 30°C. BL21(DE3) is shown for comparison. Data points are the mean of four biological replicates; error bars denote the standard deviation. Note that the absorbance values shown here are not directly comparable to those measured with a 1 cm cuvette, due to the difference in light path length.

While working with the new strains, it became obvious that by altering the outer membrane protein composition the growth behavior changed. BL21ΔABCF grew significantly more slowly than the parent strain BL21(DE3). The strain grew initially faster at 37°C than at 30°C, but the culture at 37°C saturated at a lower OD_600_ (Figure 2A). However, even at 30°C, BL21ΔABCF did not reach the same OD_600_ value as BL21(DE3).

We also observed that when using the Miller formulation of LB (with 10 g sodium chloride per liter), BL21ΔABCF grew significantly slower than in our standard LB (5 g NaCl/l), also at 30°C, whereas BL21(DE3) showed no significant differences in growth in the two media (Figure 2B). We do not currently have an explanation for this phenomenon; perhaps BL21ΔABCF is unable to compensate efficiently for the increased osmolarity of LB-Miller.

We also tested the growth of BL21ΔABCF in defined medium (Figure 2C). For this, we used minimal medium M9 supplemented with 18 amino acids. Though growth was slower than in LB, BL21ΔABCF reached higher optical densities than in the rich, undefined medium. It apparently also reached a higher density than BL21(DE3); however, part of the higher absorbance readings of the quadruple mutant cultures could be attributed to the tendency of BL21ΔABCF to clump in the defined medium, presumably due to the relatively high concentration of magnesium (2 mM; see section Aggregation of BL21ΔABCF in the Presence of Divalent Cations).

### Aggregation of BL21ΔABCF in the presence of divalent cations

**Figure 3:**
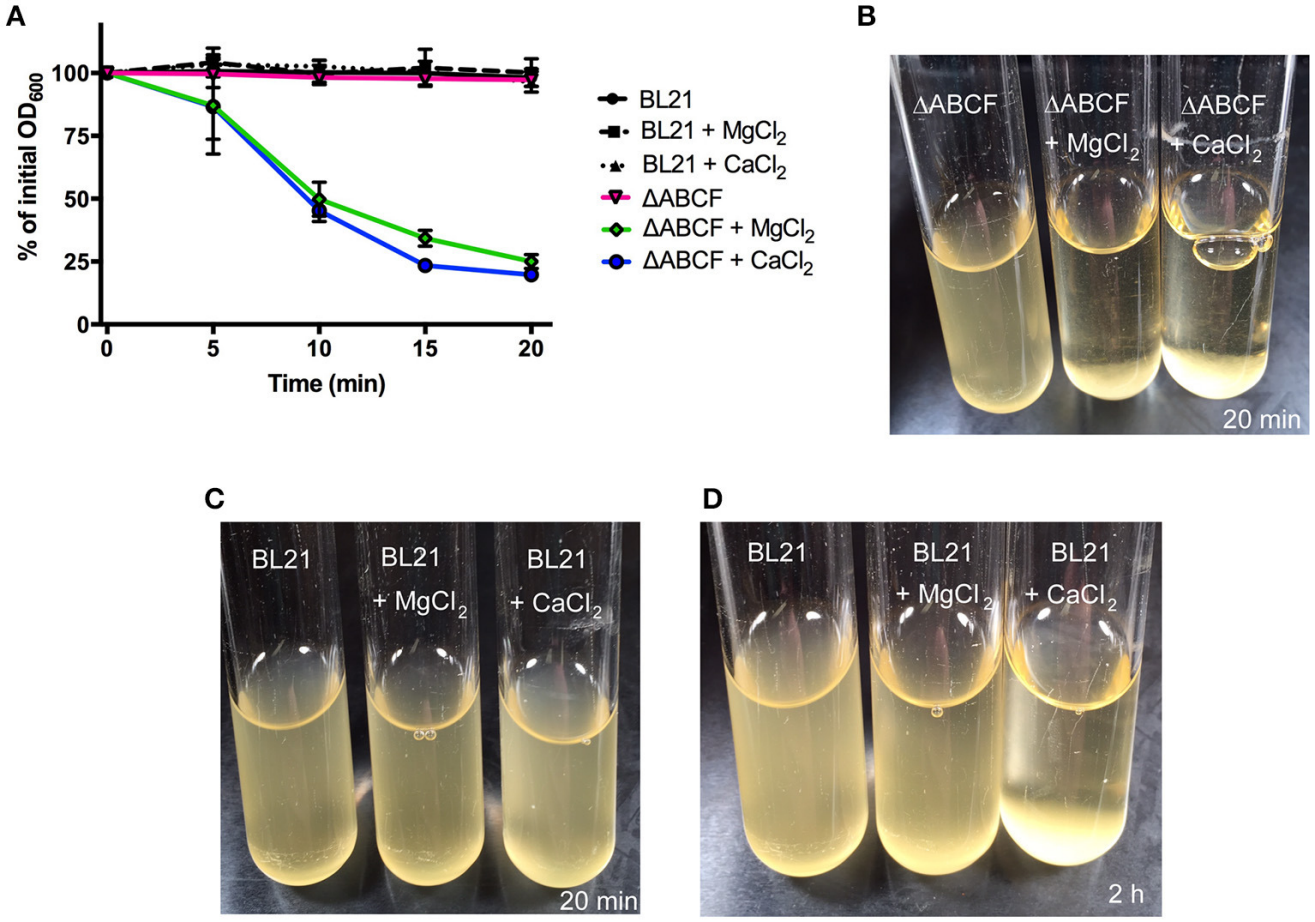
Divalent cation-mediated aggregation of BL21ΔABCF. **(A)** Quantitative sedimentation assay. Cultures of BL21ΔABCF or BL21 (DE3) were cultured in LB medium (with or without the addition of MgCl_2_ or CaCl_2_ at 10 mM) at 30°C with shaking (200 rpm). For the sedimentation assay, the cultures were incubated statically and the OD_600_ value was measured from the very top of the culture at 5-min intervals. The data points represent the percentage of the initial OD_600_ value as the mean of three biological replicates; error bars denote standard deviations. **(B)** Photograph of BL21ΔABCF cultures after 20 min of static incubation. In the presence of 10 mM MgCl_2_ or CaCl_2_, BL21ΔABCF flocculates and rapidly settles at the bottom of the tube, leaving the medium clear. **(C)** Photograph of BL21(DE3) cultures after 20 min of static incubation. Turbidity is not reduced by the addition of 10 mM MgCl_2_ or CaCl_2_ to cultures of BL21(DE3). **(D)** Photograph of BL21(DE3) cultures after 2 h of static incubation. In the presence of CaCl_2_, also BL21(DE3) flocculates.

As noted above, BL21ΔABCF tends to aggregate in the presence of divalent cations. When Mg^2+^ or Ca^2+^ is added to the medium, BL21ΔABCF flocculates and settles rapidly at the bottom of the tube under static condition (Figures 3A,B). In contrast, the parent strain does not aggregate in the presence of either ion during the short time frame of the experiment (Figures 3A,C). However, upon prolonged incubation (>1 h), also BL21(DE3) began to flocculate in the presence of Ca^2+^ (Figure 3D). The reason for the rapid aggregation of BL21ΔABCF is not clear. A possibility might be the increased binding of divalent cations by the more exposed lipid A phosphates in the BL21ΔABCF strain, leading to electrostatic attraction through bridging divalent cations. In principle, the aggregation caused by CaCl_2_ might cause problems when preparing chemically competent cells; however, in our hands BL21ΔABCF can be made competent and transformed efficiently using the standard CaCl_2_ protocol (data not shown).

### BL21ΔABCF is superior in producing recombinant OMPs

To qualitatively test the performance of our new quadruple mutant strain, we over-expressed four test proteins from our laboratory. For comparison, we used the parent strain *E. coli* BL21 (DE3). The test proteins were OmpX, a native OMP of *E. coli*, a duplicated variant of this protein with 16 β-strands rather than the usual eight (Arnold et al., 2007), and another OmpX variant containing a HA tag in one of the extracellular loops. Additionally, we tested the expression of two autotransporter proteins: the membrane anchor domain of the *Yersinia* adhesin YadA (Wollmann et al., 2006) and the inverse autotransporter Intimin from enteropathogenic *E. coli* (Oberhettinger et al., 2012).

**Figure 4:**
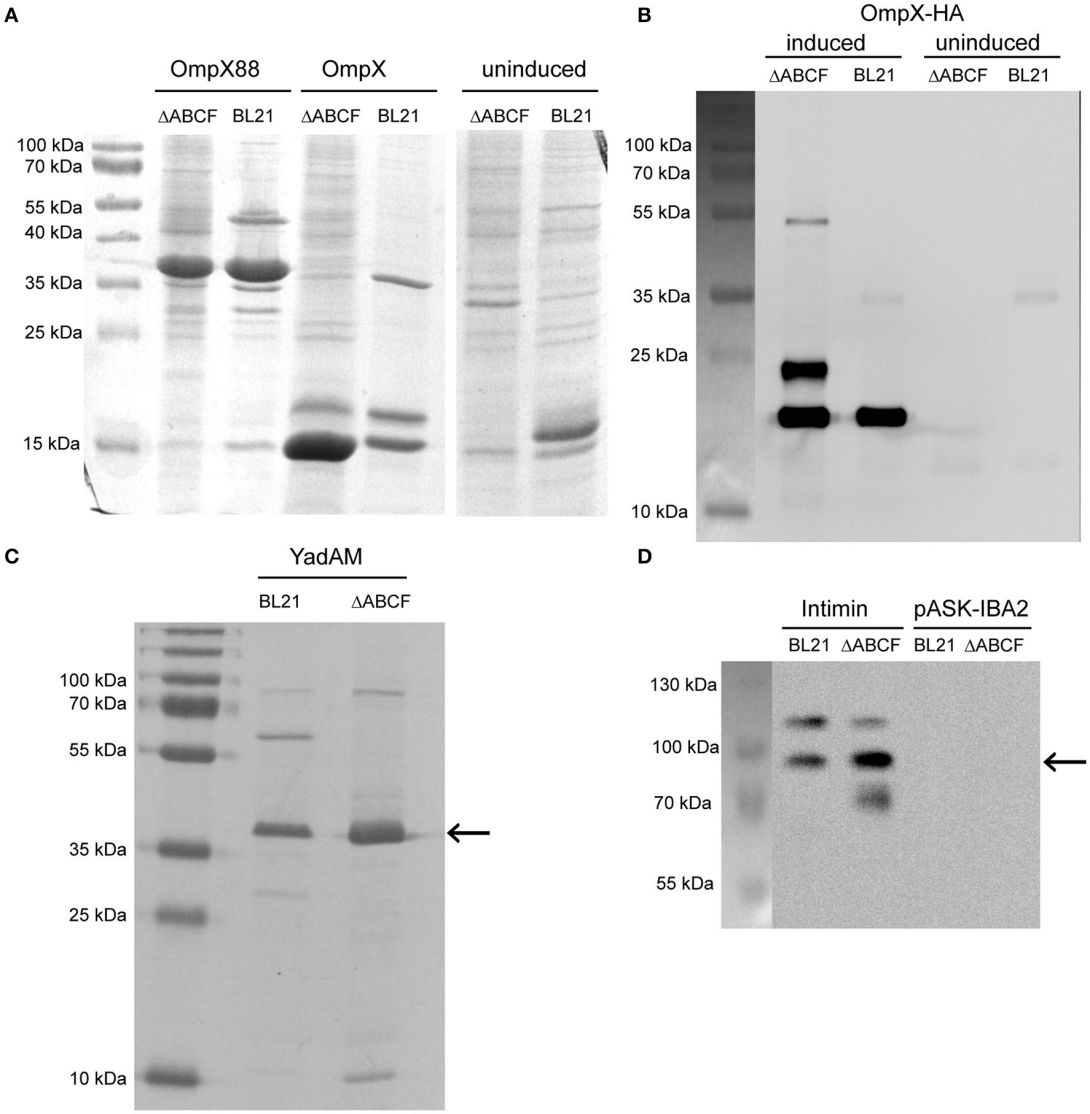
Improved over-production of recombinant OMPs in BL21ΔABCF. **(A)** Coomassie-stained 15% polyacrylamide gel showing production levels of recombinant OmpX and duplicated OmpX (OmpX88) in BL21(DE3) and BL21ΔABCF. Uninduced cultures of the OmpX construct are shown as a control. Note that the artificial construct OmpX88 does not show significantly improved expression, while the expression of OmpX is massively improved in the BL21ΔABCF strain. **(B)** Western blot of OmpX-HA produced in both BL21(DE3) and BL21ΔABCF probed with an anti-HA antibody. An equal amount of cells (based on OD_600_ measurement) was lysed by heating in sample buffer and loaded onto the gel. Uninduced samples are shown as controls. A picture showing the positions of the pre-stained molecular weight marker bands on the blotting membrane is shown on the left. **(C)** Colloidal Coomassie G250-stained 15% polyacrylamide gel showing production levels of the YadA membrane anchor (YadAM; position denoted by the arrow) produced in both BL21 and BL21ΔABCF. **(D)** Western blot of Intimin produced in both BL21 and BL21ΔABCF probed with an anti-Intimin antibody. An equal amount of cells (based on OD600 measurement) was lysed by heating in sample buffer and loaded onto the gel. Strains with the empty vector (pASK-IBA2) serve as controls. The arrow shows the position of the main Intimin band (~95 kDa). A picture showing the positions of the pre-stained molecular weight marker bands on the blotting membrane is shown on the left.

The expression of wild-type OmpX can be seen as a band at 15 kDa when stained with Coomassie G-250 (Figure 4A). The band is more intense for BL21ΔABCF than for the parent strain. Other bands of native proteins, for example the band at 18 kDa that presumably represents OmpW, show that the same amount of sample was loaded. Note that there is no clear difference between the strains for the duplicated variant OmpX88 (~37 kDa). The OmpX-HA construct was also expressed in BL21(DE3) and BL21ΔABCF. A Western blot of outer membrane preparations (Figure 4B) shows that for the quadruple knock-out strain, the total amount of expressed OmpX-HA is higher compared to the parent strain BL21(DE3). An additional band was detected at an increased molecular weight (~25 kDa) that we originally attributed to non-denatured OmpX-HA (Figure 4B, right panel), similar to previous findings on OmpX gel shifts (Arnold et al., 2007), Based on the OMP gel shift phenomenon (Rosenbusch, 1974; Schweizer et al., 1978), we know that native and denatured forms of OMPs can migrate differently in SDS-PAGE. However, when comparing heated and unheated samples, the band did not change, suggesting that it is an artifact yet to be explained (data not shown). The third specific band at ~50 kDa is presumably a folded dimer of OmpX at very low concentration (Chaturvedi and Mahalakshmi, 2013).

YadA is an obligate homotrimer and an adhesin of enteropathogenic *Yersiniae* (Mühlenkamp et al., 2015). It is an extremely stable protein which remains trimeric in the presence of denaturants such as, SDS and urea (Wollmann et al., 2006). In SDS-PAGE, YadAM (membrane anchor domain of YadA) migrates at an apparent molecular weight of 45 kDa (Wollmann et al., 2006). The expression of YadAM in BL21(DE3) and BL21ΔABCF is shown in Figure 4C. The colloidal Coomassie G250-stained gel shows better expression of YadAM in BL21ΔABCF than BL21(DE3). As mentioned above, many native outer membrane protein bands (e.g., ~30 kDa and ~60 kDa) are absent in the ΔABCF strain.

The expression of Intimin is shown in Figure 4D. Here, a construct including a StrepII tag was used and its expression visualized specifically in a Western blot using an anti-Intimin antibody (Oberhettinger et al., 2012). The blot shows two bands for the parent strain: one at ~95 kDa corresponding to the molecular weight of Intimin and a second band at ~120 kDa. This latter band is sometimes observed in Intimin blots, though its provenance is not clear (Heinz et al., 2016; Leo et al., 2016). For the ΔABCF strain, the 95 kDa band is more intense, and the ~120 kDa band fainter, suggesting better membrane insertion. In addition, some apparent degradation product can be seen for the quadruple mutant (band at ~70 kDa). The superiority of the BL21ΔABCF strain is demonstrated by a higher yield of the “correct” Intimin band and less of the (presumably mis-incorporated) 120 kDa band.

To gain a more quantitative view of OMP production in BL21ΔABCF and to assess reproducibility between culture batches, we performed whole-cell ELISA on bacteria expressing OmpX-HA and Intimin. We compared expression of these two proteins in BL21(DE3), BL21ΔABCF, and the Prilipov strain BL21 Omp8. We also tested the expression of YadAM in these strains, but due to technical problems with detecting the StrepII tag on the bacterial surface combined with the tendency of YadAM-expressing cells to clump, we did not obtain reliable results (data not shown). In this construct, only a short stretch of YadAM is exposed to the surface, so the StrepII tag is presumably not fully accessible to antibodies (Shahid et al., 2012).

**Figure 5:**
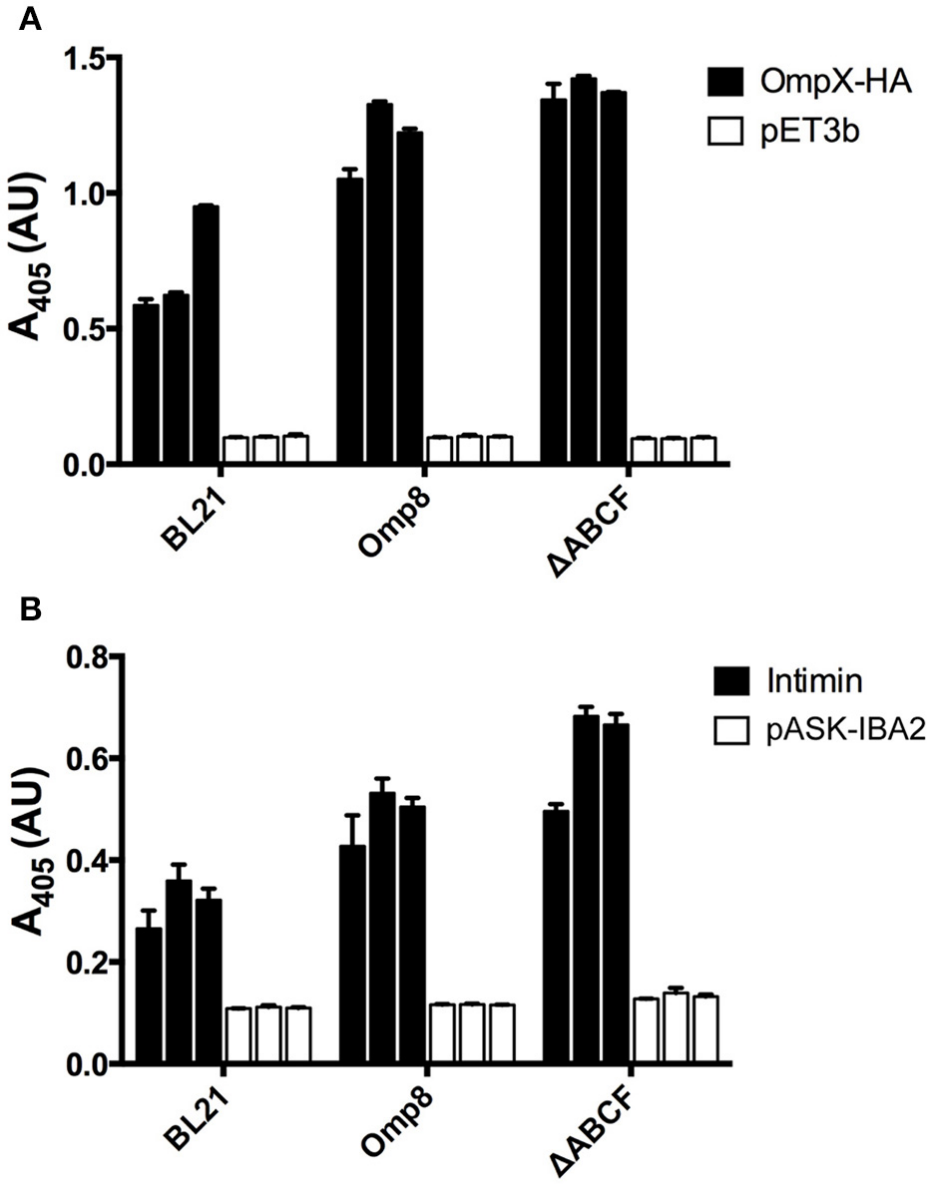
Quantitative assessment of recombinant OMP production by BL21ΔABCF **(A)** Whole-cell ELISA of bacteria producing OmpX-HA. Bacteria were coated onto wells of a microtiter plate and probed with an anti-HA tag antibody. The primary antibody was detected with a HRP-conjugated secondary antibody and colorimetric staining using the substrate ABTS. Black bars denote the biological replicates (three for each strain) expressing OmpX-HA; white bars show values for empty vector controls. Each bar represent the mean of three technical replicates; error bars denote the standard deviation of three technical replicates. **(B)** Whole-cell ELISA of bacteria producing Intimin. The experimental set up was similar to **(A)**; the primary antibody was an anti-Intimin antibody. Black bars denote biological replicates expressing Intimin, white bars represent vector controls. Each bar represent the mean of three technical replicates; error bars denote the standard deviation of three technical replicates.

BL21ΔABCF produced both Intimin and OmpX-HA at higher amounts than the parent strain BL21(DE3) (Figure 5). Somewhat unexpectedly, BL21ΔABCF also outperformed the Omp8 strain, though the difference is not large. The ELISA also demonstrate that the variability between biological replicates is low, though for Intimin there is more variability between replicates. Incidentally, in the whole-cell assays the detected proteins must be surface-exposed, showing that the detected species must be correctly processed and inserted into the membrane.

## Discussion

We have produced a series of *E. coli* knock-out strains for use in over-expressing OMPs with deletions of at least one of four abundant OMP protein genes. Our series contains the four single deletions, all combinations of double and triple deletions, and the quadruple deletion strain BL21ΔABCF. The strains all contain the DE3 lysogen and can therefore be used with vectors requiring the T7 polymerase for expression.

We noted some unusual properties when culturing the quadruple mutant strain BL21ΔABCF: the strain grows poorly at 37°C and does not tolerate high salt concentrations. In addition, BL21ΔABCF aggregates in the presence of divalent cations. We therefore recommend that BL21ΔABCF be grown at 30°C in medium with low sodium chloride (≤5 g/l) and without excess divalent cations.

We demonstrated the superiority of the quadruple mutant strain in producing four different test proteins (OmpX, artificial OmpX variants, YadA, and Intimin) compared with BL21(DE3). This strain has mutations in the same genes as the Omp8 strain previously produced (Prilipov et al., 1998b), and we assumed that there would be no major differences between these two strains regarding OMP production capability. However, our whole-cell ELISAs showed that BL21ΔABCF is slightly better at over-expressing OMPs than Omp8, and significantly better than the parent strain BL21(DE3). In addition, as our strain lacks the transposon found in Omp8, and the full deletion of the OMP coding sequences prevents any reversion to wild-type, it is more stable than the Omp8 strain has proven to be, at least in our hands. A second advantage of the ΔABCF strain is the lack of any intrinsic antibiotic resistance markers, allowing it to host a broader choice of vector plasmids.

An additional advantage of these OMP deletion strains, similarly to the strains of Prilipov et al., is the low level of endogenous OMPs. Especially the ΔABCF strain can be used for *in situ* studies of OMP functions, without interference from endogenous proteins, where efficient labeling of the protein of interest against a low background is required. The power of such approaches can be seen in work where YadA was expressed in the original Omp8 strain in isotope-labeled medium for nuclear magnetic resonance (NMR) studies, where it was then possible to directly measure NMR spectra of the protein in native membranes (Shahid et al., 2015). This would not have been possible using wild-type *E. coli* due to the high background from other abundant OMPs.

Furthermore, the lack of all major naturally occurring OMPs in this strain may aid in purifying heterologous OMPs for functional or structural studies. As the amount of competing OMPs is low, heterologous OMPs can be purified efficiently and simply with e.g., ion exchange chromatography, without the need to introduce affinity tags, which might compromise protein function. This applies even to transmembrane β-barrel proteins of eukaryotic origin, some of which have been produced in bacteria (Walther et al., 2009a).

## Strain availability

All strains produced in this study are available through Addgene (http://www.addgene.org). See Table 2 for strain identifiers.

## Author contributions

DL conceived the project; JL and DL designed the project; IM and JL performed the recombinant DNA work; IM, JL, MM, and NC performed the strain characterization; and all authors were involved in analyzing the data and writing the manuscript.

### Conflict of interest statement

The authors declare that the research was conducted in the absence of any commercial or financial relationships that could be construed as a potential conflict of interest. This work has been included in a patent application (U.S. Patent Office application number 62438094).
