# Corrigendum: A New Strain Collection for Improved Expression of Outer Membrane Proteins

**DOI:** 10.3389/fcimb.2020.00220

**Published:** 2020-06-03

**Authors:** Ina Meuskens, Marcin Michalik, Nandini Chauhan, Dirk Linke, Jack C. Leo

**Affiliations:** ^1^Section for Evolution and Genetics, Department of Biosciences, University of Oslo, Oslo, Norway; ^2^Interfaculty Institute for Biochemistry, Eberhard Karls University, Tübingen, Germany

**Keywords:** outer membrane, β-barrel protein, recombinant protein expression, P1 transduction, production strain

In the original article, there was an error. In contrast to what was stated in the paper, the deletions were made in *E. coli* BL21 Gold (DE3), not in BL21(DE3). This does not have consequences for the validity of the research conducted, or for the use of the strains for overproduction of heterologous OMPs. However, due to the different genetic background of the strains, the deletion mutants display resistance against tetracycline and have an enhanced transformation efficiency. The authors would like to thank David Six at VinatoRx Pharmaceuticals, Inc. for bringing this to their attention.

A correction has been made to the entire article. Every reference to “BL21(DE3)” as the parent strain, has been replaced with “BL21 Gold (DE3).” Our strains are derivatives of BL21 Gold (DE3), but the control strain used was BL21(DE3). In addition, the legends for Figures 1–3 and Table 2 have been updated and appear below.

“Figure 1. Verification of the BL21 OMP knock-out strains. **(A)** Colony PCR results with primers for *ompA, ompF*, and *lamB* showing that in the mutant strains only a scar sequence (130–150 bp) remains at the locus. BL21 = (DE3) control (expected sizes: *ompA* 1072, *ompF* 1135, *lamB* 1380 bp). **(B)** Colony PCR results of *ompC*. On the left, results using primers specific for the K-12 *ompC* locus are shown, where BL21(DE3) does not give a product and the deletion strains show just a short scar sequence (~150 bp). The expected size for K-12 *ompC is* 1101 bp. On the right, results using common primers amplifying a larger region around the *ompC* locus in both BL21 and K-12. Here, the expected product for BL21(DE3) is 1.9 kb, the size expected for *E. coli* K-12 product is 5.3 kb and for the deletion strains 4.2 kb. **(C)** Silver-stained 15% polyacrylamide gel of BL21 OMP knock-out strains. The positions of OmpA (black arrowhead) and OmpC/F (open arrowhead) bands are indicated for the control (BL21). LamB is poorly expressed in *E. coli* B strains when grown at temperatures above 30°C and in the presence of other carbon sources (Ronen and Raanan-Ashkenazi, 1971), so this protein is not evident in most of the samples. Note that the Δ symbol has been omitted in the figure texts due to space constraints.”

“Figure 2. Growth properties of the quadruple mutant BL21ΔABCF. **(A)** Growth of BL21ΔABCF in LB medium at 30 and 37°C. BL21(DE3) is shown for comparison. Data points are the mean of four biological replicates; error bars denote the standard deviation. **(B)** Growth of BL21ΔABCF in LB (0.5% NaCl) and LB-Miller (1% NaCl) medium at 30°C. BL21(DE3) is shown for comparison. Data points are the mean of four biological replicates; error bars denote the standard deviation. **(C)** Growth of BL21ΔABCF in supplemented M9 medium at 30°C. BL21(DE3) is shown for comparison. Data points are the mean of four biological replicates; error bars denote the standard deviation. Note that the absorbance values shown here are not directly comparable to those measured with a 1 cm cuvette, due to the difference in light path length.”

“Figure 3. Divalent cation-mediated aggregation of BL21ΔABCF. **(A)** Quantitative sedimentation assay. Cultures of BL21ΔABCF or BL21 (DE3) were cultured in LB medium (with or without the addition of MgCl_2_ or CaCl_2_ at 10 mM) at 30°C with shaking (200 rpm).”

“Table 2. Knock-out strains produced in this study. All strains are derived from BL21 Gold (DE3). With the genotype: *E. coli* B F^−^ *ompT hsdS*(rB^−^ mB^−^) *dcm*^+^ Tet^r^ *gal endA* Hte.”

The authors apologize for these errors and state that this does not change the scientific conclusions of the article in any way. The original article has been updated.
