# Supplementary material for: A New Strain Collection for Improved Expression of Outer Membrane Proteins
Source: Front Cell Infect Microbiol. 2017 Nov 7;7:464. doi: 10.3389/fcimb.2017.00464 (PMC5681912; doi:10.3389/fcimb.2017.00464)
Supplement: Supplementary file 1 [file Image1.PDF]

Meuskens *et al*., A New Strain Collection for Improved Expression of Outer Membrane Proteins

Supplementary Figure 1

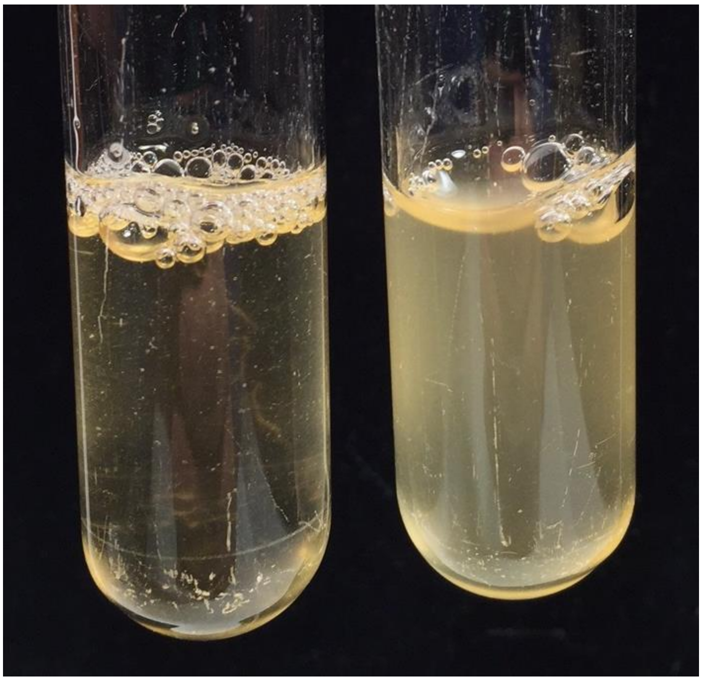

Spontaneous lysis of BL21 Omp8. In our hands, BL21 Omp8 has proven to be unstable and prone to spontaneous lysis. However, this phenomenon appears to be stochastic and we cannot predict when this will occur. In a set of 25 cultures of BL21 Omp8 harbouring the pIBA2-YadAM plasmid, one culture lysed upon induction with anhydrotetracycline (left). An unlysed control is shown for comparison on the right.
